# Clinical, radiological, and genetic characterization of *SLC13A5* variants in Saudi families: Genotype phenotype correlation and brief review of the literature

**DOI:** 10.3389/fped.2022.1051534

**Published:** 2023-02-27

**Authors:** Hanan AlQudairy, Hesham AlDhalaan, Sarah AlRuways, Nouf AlMutairi, Maha AlNakiyah, Reema AlGhofaili, Albandary AlBakheet, Adeeb Alomrani, Omar A. Alharbi, Ehab Tous, Moeen AlSayed, Hamad AlZaidan, Maha M. AlRasheed, Ali AlOdaib, Namik Kaya

**Affiliations:** ^1^ Translational Genomic Department, Center for Genomic Medicine, King Faisal Specialist Hospital, and Research Centre (KFSHRC), Riyadh, Saudi Arabia; ^2^ Department of Neurosciences, KFSHRC, Riyadh, Saudi Arabia; ^3^ College of Pharmacy, King Saud University (KSU), Riyadh, Saudi Arabia; ^4^ Dentistry Department, KFSHRC, Riyadh, Saudi Arabia; ^5^ Department of Medical Genomics, Center for Genomic Medicine, KFSHRC, Riyadh, Saudi Arabia; ^6^ Department of Clinical Pharmacy, College of Pharmacy, King Saud University, Riyadh, Saudi Arabia; ^7^ Training and Education Department, Research Centre, KFSHRC, Riyadh, Saudi Arabia

**Keywords:** *SLC13A*, novel variant, epileptic encephalopathy, tooth abnormalities, intellectual disability, whole exome sequencing (WES), Sanger sequencing

## Abstract

**Background:**

*SLC13A5* (solute carrier family 13, member 5) encodes sodium/citrate cotransporter, which mainly localizes in cellular plasma membranes in the frontal cortex, retina, and liver. Pathogenic variants of the gene cause an autosomal recessive syndrome known as “developmental and epileptic encephalopathy 25 with amelogenesis imperfecta.”

**Results:**

Here, we have investigated six patients from three different consanguineous Saudi families. The affected individuals presented with neonatal seizures, developmental delay, and significant defects in tooth development. Some patients showed other clinical features such as muscle weakness, motor difficulties, intellectual disability, microcephaly, and speech problems in addition to additional abnormalities revealed by electroencephalography (EEGs) and magnetic resonance imaging (MRI). One of the MRI findings was related to cortical thickening in the frontal lobe. To diagnose and study the genetic defects of the patients, whole exome sequencing (WES) coupled with confirmatory Sanger sequencing was utilized. Iterative filtering identified two variants of *SLC13A5*, one of which is novel, in the families. Families 1 and 2 had the same insertion (a previously reported mutation), leading to a frameshift and premature stop codon. The third family had a novel splice site variant. Confirmatory Sanger sequencing corroborated WES results and indicated full segregation of the variants in the corresponding families. The patients’ conditions were poorly controlled by multiple antiepileptics as they needed constant care.

**Conclusion:**

Considering that recessive mutations are common in the Arab population, *SLC13A5* screening should be prioritized in future patients harboring similar symptoms including defects in molar development.

## Introduction

SLC13 genes (*SLC13A2* and *SLC13A3*) encode transporter proteins that carry Na+-coupled dicarboxylate (NaDC) across the plasma membrane with different substrate preferences (1). Unlike NaDC, *SLC13A5* encodes a Na+-coupled citrate (NaCT) transporter protein, a cytoplasmic sodium-dependent citrate carrier, which can carry dicarboxylate in addition to tricarboxylate substrates (2). The transporter is widely expressed in neurons, localized in the plasma membrane of various cell types, including hepatocytes in the liver, spermatozoa in the testis, and mostly astrocytes and neurons in the brain (3). Citrate is vital in cellular metabolism and neurotransmitter biogenesis (3). It is known to have an important role in the tricarboxylic acid cycle, where the molecule represents the starting point for generating reducing equivalents nicotinamide adenine dinucleotide (NADH) and flavin adenine dinucleotide (reduced form) (FADH2), which in turn enter the electron transport chain to generate ATP (4). The brain cannot produce citrate independently; hence, it depends on citrate uptake *via* NaCT. Thus, the carrier has a pivotal role in mediating the uptake of circulating citrate for metabolism (4), preferably in the trivalent form rather than the divalent form.

*SLC13A5* is located on chromosome 17 and consists of 12 coding exons. Biallelic mutations in the gene cause a rare genetic syndrome known as “developmental and epileptic encephalopathy 25 with amelogenesis imperfecta” (DEE25; phenotype MIM: 615905). The mutant NaCT molecules can lose their ability to bind to the sodium molecules, therefore failing to transport citrate from the extracellular matrix to cytosol (3, 5, 6). Relatedly, when citrate transport and metabolism are disrupted, intracellular citrate levels fall, resulting in neuronal energy failure, which is thought to be one of the explanations behind epileptic symptoms (3). In other words, a lack of cellular citrate results in energy deficiency in the brain, thus possibly contributing to the pathogenesis of epilepsy and delayed brain development (6, 7).

Here, in this study, we investigate pathogenic homozygous variants of *SLC13A5* (including a novel splice site variant), causing mainly neonatal seizures and developmental delay in six patients from three different Saudi families. The affected individuals also showed auxiliary clinical features such as muscle weakness, motor difficulties, intellectual disability, microcephaly, and speech problems, in addition to abnormalities revealed by electroencephalography (EEG). The patients’ conditions were poorly controlled by multiple antiepileptics as they needed constant care. Additionally, we reviewed the current literature about *SLC13A* variants.

## Materials and methods

### Patient recruitment, sample collection, and nucleic acid isolation

Six patients and related family members were recruited under approvals provided by Institution's Research Advisory Council (Ethics Statement) at King Faisal Specialist Hospital and Research Center, Riyadh, KSA (KFSHRC, IRB-approved protocols, RAC#2120022). Peripheral blood samples (5 ml) were drawn into EDTA tubes. The Gentra Puregene DNA Purification Kit was utilized during DNA isolation (Gentra Systems, Inc. Minneapolis, MN, United States).

### PCR and Sanger sequencing-based variant screening

Gene-specific primers were designed using the Primer 3 web tool. Primers were tested on human control DNA samples and optimized for successful PCR amplifications. After the PCR, samples were purified and sent to the sequencing core facility for Sanger sequencing. The direct sequencing was done on an ABI PRISM 3100 Genetic Analyzer (Applied Biosystems, Foster City, CA, United States) according to the manufacturer's instructions.

### Genome-wide SNP screening using GeneChip Axiom assays and autozygosity analysis

Genotyping was undertaken using GeneChip Human Axiom Arrays and other equipment such as a hybridization oven and a titan platform from Affymetrix (Thermo Fisher Inc., Santa Clara, CA, United States). Sample processing, labeling, hybridization, washing, and scanning were all done according to manuals and assay protocols provided by the manufacturers.

### Whole exome sequencing

Exomes were captured using the Sure Select kit (Agilent Technologies, Santa Clara, CA, United States). Library construction was done using the same kits (Exons V5, 50 Mb capture kit, Agilent Technologies). After library preparation, captured fragments were run on an Illumina HiSeq 2500 Sequencer and mapped against UCSC hg19 (Illumina, Inc., San Diego, CA, United States). Comprehensive filtering of the detected variants was done as previously published (8–11). Especially, variants based on homozygosity, coding, and splicing features, being within the autozygome of the affected individuals in the families, were prioritized during filtering analysis. Additionally, publicly available databases and local resources, such as the program for Saudi Human Genome-based data outputs, were also screened during filtering. In silico pathogenicity was carried out as reported before (8–11).

## Results

### Clinical features

#### Patient 1 (family 1, IV:3)

**Figure 1:**
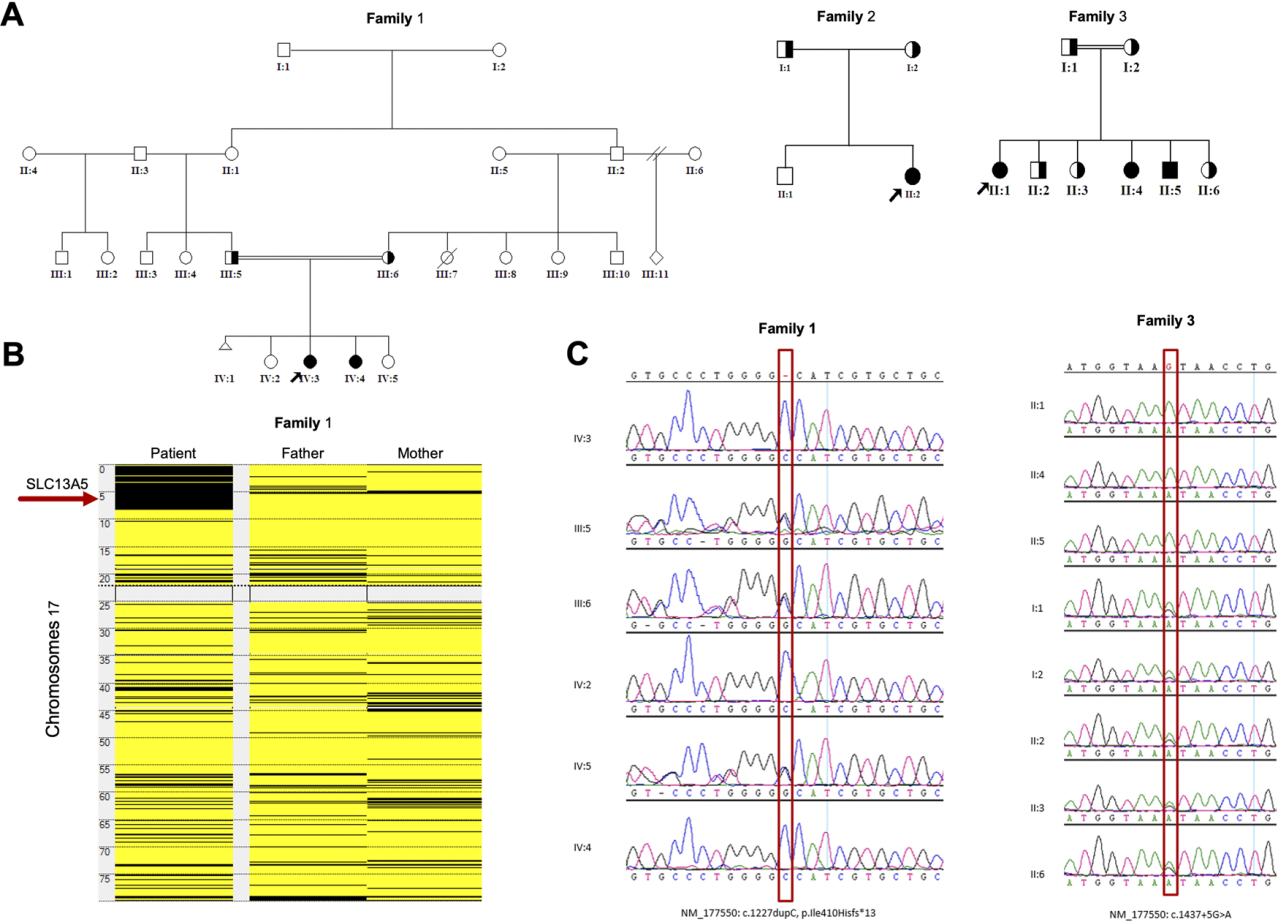
Genetic analyses of the families: (**A**) Pedigrees of the families of the study showing six affected members. (**B**) Homozygosity mapping showing the location of the *SLC13A5* gene in major ROH in family 1. (**C**) Sanger sequence chromatograms of the tested individuals. *SLC13A5*, solute carrier family 13, member 5; ROH, runs of homozygosity.

The patient is a 17-year-old girl from a Saudi consanguineous family with a positive family history of a similar medical condition (Figure 1A). The patient developed a seizure on the first day of her life but was only seen in the clinic at the age of 8 years with a history of developmental delay and seizures mainly described in the form of eye fluttering. According to her parents, she started crawling, standing, and sitting at the age of 11 years but was unable to walk. On examination, all her growth parameters were below the third percentile with microcephaly. Microcephaly was detected in a local hospital when she was 4 months old. Neurological examination showed spastic quadriplegia, with contracture and increased deep tendon reflexes. She was on levetiracetam (equivalent to 66 mg/kg/day), phenobarbital (equivalent to 7 mg/kg/day), and topiramate (equivalent to 7 mg/kg/day) and was free of seizure for more than 2 years. At the age of 14, she suffered a femur fracture without any clear history of trauma and developed a seizure. The seizure was in the form of generalized tonic posturing with up-rolling of the eyes with involuntary movements. It lasted for 1 min and aborted spontaneously. Her GAL-1P levels were less than 49, and this result was consistent with the diagnosis of a very mild form of Duarte galactosemia. EEG was done twice at 8 and 14 years of age, and it showed intermittent slow activity, maximum in the left temporal, with excessive fast activity that could be due to phenobarbital (a chronic medication used to treat the patient during that time).

#### Patient 2 (family 1, IV:4)

The patient is a 14-year-old girl, the sister of patient 1 (Figure 1A), diagnosed with developmental delay and seizure disorder. Her history of seizures began after 12 h of birth. She suffered from seizures many times per day. Her seizures were controlled by phenobarbitone for a few months only. Afterward, topiramate was added to her medication, which reduced the frequency of her seizures (3–20 times/day) per day. At 15 months, she was neither able to support herself nor was she attempting to stand. The last EEG done when the patient was 1 year old showed mild abnormality, probably secondary to a medication's effect.

#### Patient 3 (family 2, II:2)

The patient is a 7-year-old girl (Figure 1A). She started to have symptoms on her first day of life. The patient presented to the clinic at the age of 9 months and was diagnosed with severe infantile epileptic encephalopathy, global developmental delay (GDD), and intractable epilepsy disorder. Currently, the patient is unable to walk, crawl, or sit without support, and lacks control of her head and neck. Her seizures had multiple semiologies with left-sided facial twitches and secondary generalization with generalized tonic and clonic seizures. Another type of seizure was also noticed as right-sided tonic flexion of the extremities and extension of the left side accompanied by secondary generalization followed by postictal incontinence, both urinary and bowel, as well as sleepiness and confusion. At the age of 3, her seizures have been controlled for 1 year, though previously occurring on a daily basis. Since her birth and during the disease course, the patient was on multiple medications to control her seizures (e.g., phenobarbital, levetiracetam, carbamazepine, valproic acid, topiramate, clobazam) with poor response and sometimes no response. EEG done at the age of 9 months showed diffuse encephalopathy, which might be due to medications or postictal status. Subsequent EEG examinations at the age of 2 and 3 years were all normal. However, the last EEG at the age of 6 years was abnormal and suggestive of focal epileptogenicity from the left occipitotemporal head region. Lab tests done during the last visit revealed ammonia levels higher than normal.

#### Patient 4 (family 3, II:1)

The patient is a 28-year-old woman with a positive family history of two affected siblings with similar phenotypes, a 17-year-old sister with a more severe condition (patient 5) and a 12-year-old brother with a mild condition (patient 6) (Figure 1A). Her seizures started on the second day of life with abnormal EEG showing epileptiform discharges. Her seizures were controlled with valproic acid, and her last EEG was normal. She also has GDD. She can speak a few words. She started to walk at the age of 4 years. Her IQ was below 50. She is on risperidone for anxiety and aggressiveness. She is wearing eyeglasses for divergent squint. Her brain MRI (magnetic resonance imaging) and MRA (magnetic resonance angiography) are normal.

#### Patient 5 (family 3, II:4)

**Figure 2:**
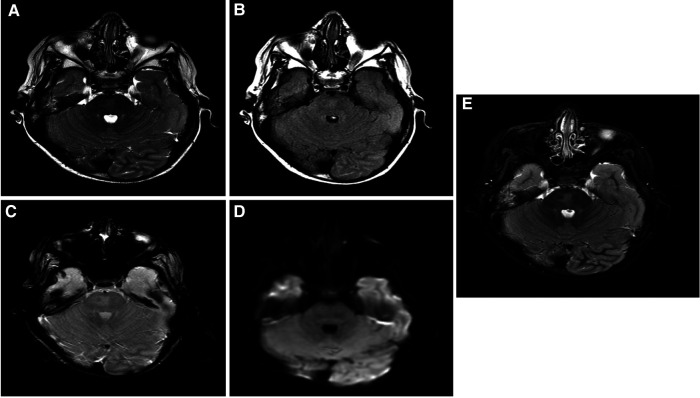
Brain MRI findings: selected images at the level of superior cerebellar peduncles showing a mildly increased signal intensity on a T2-weighted image (**A**), FLAIR (**B**), and inversion recovery (**C**) without evidence of blood staining on a T2 star image (**D**) or an increased diffusion signal (**E**). These findings are most suggestive of nonspecific pontine gliosis. MRI, magnetic resonance imaging; FLAIR, fluid-attenuated inversion recovery.

The patient is a 17-year-old woman (Figure 1A). Her first seizure started when she was a neonate (Table 1). She first presented in our hospital at the age of 4 years with infantile epileptic encephalopathy, severe global developmental delay, neonatal seizures, microcephaly, and hypertonia. She was noticed to have microcephaly when she was 1 month old. Her seizures started with left-sided body tonic posture and versive head deviation to the left, evolving to secondary generalization and myoclonic attacks. The first EEG was done at the age of 4, which showed severe abnormalities with the presence of a focal area of cortical neuro irritability on the right central and left parietal regions, indicating a predisposition for epileptic seizures and focal slowing on the right parasagittal region, which suggested an underlying structural abnormality. The last EEG was done at the age of 11 years. The finding was suggestive of mild cortical dysfunction and nonspecific encephalopathy. MRI done at the age of 3 years showed a mildly increased signal intensity on a T2-weighted image (Figure 2A). The patient is still having frequent seizures, is bedridden, is totally dependent on her family, and is taking multiple antiepileptic medications.

**Table 1:** Clinical features of subjects reported in this study.

Family #		1		2	3		
Subject		1	2	3	4	5	6
Pedigree number		IV:3	IV:4	II:2	II:1	II:4	II:5
Gender		Female	Female	Female	Female	Female	Male
Age of onset		First day of life	Around 12 h after birth	First day of life	Second day of life	Neonate	First day of life
Outcome (alive or dead)		Alive	Alive	Alive	Alive	Alive	Alive
If alive current age		17 years	14 years	6 years	28 years	17 years	12 years
Consanguinity		(+)	(+)	(+)	Same tribe	Same tribe	Same tribe
Ethnicity		Arab (Saudi)	Arab (Saudi)	Arab (Saudi)	Arab (Saudi)	Arab (Saudi)	Arab (Saudi)
Family history of similar illness		(+)	(+)	(−)	(+)	(+)	(+)
Mutation	c.DNA change	c.1227dupC	c.1227dupC	c.1227dupC	c.1437 + 5G > A	c.1437 + 5G > A	c.1437 + 5G > A
	Amino acid change	*p*.I410H*13	*p*.I410H*13	*p*.I410H*13			
	Mutation type	Frameshift duplication	Frameshift duplication	Frameshift duplication	Splicing	Splicing	Splicing
	Zygozity	Homozygous	Homozygous	Homozygous	Homozygous	Homozygous	Homozygous
Neurological findings	Global developmental delay (GDD)	(+)	(+)	(+)	(+)	(+)	(+)
	Muscle weakness	NA	NA	(+)	(+)	(+)	NA
	Motor difficulties	(+)	(+)	(+)	(+)	(+)	(−)
	Hypotonia	NA	(+)	NA	NA	(+)	NA
	Seizure	(+) Mainly described in the form of eye fluttering; after being controlled with medication for more than 2 years, she developed seizures in the form of generalized tonic posturing and up-rolling of the eyes for 1 min, which was aborted spontaneously	(+) Convulsions	(+) Multiple Semiologies	(+)	(+) Actually starts with left-sided body tonic posture and versive head deviation to the left, evolving to secondary generalization and myoclonic attacks	(+)
	Ataxia	NA	NA	NA	NA	(−) Bedridden	NA
	Intellectual disability	(+)	NA	NA	NA	(+)	(+)
	Speech problems	(+)	(+)	NA	She can speak a few words	(+)	(+)
	Hearing impairment	(−)	NA	NA	NA	NA	NA
	Spasticity	(+)	NA	NA	NA	(+)	NA
	Abnormal involuntary movements	(+)	NA	NA	NA	NA	NA
	Others	Spastic quadriplegia				Appendicular hypertonia	
Neuroimaging	MRI	Normal	Normal	Unremarkable apart from mild enlargement of subarachnoid spaces and ventricular system	Normal	Focal cortical dysplasia, minimal cortical thickening in the frontal lobe	NA
	MRS	(−)	(−)	NA	NA	(+) Lactate and glutamate in the first visit only	NA
	EEG	Done at the age 8 and 14 years and showed intermittent slow activity, maximum in the left temporal with an excessive fast activity that could be due to phenobarbital, a chronic medication the patient is taking	Mild abnormalities are probably due to medication effect	Done at the age of 9 months and showed diffuse encephalopathy that might be due to medications or postical status; others EEG examinations at the age of 2 and 3 years were all normal. The last EEG at the age of 6 years was abnormal and suggestive of focal epileptogenicity from the left occipitotemporal head region	Done on the second day of life after her first seizure and showed epileptiform discharges; now, her seizures re controlled valproic acid, with the last EEG being normal.	First EEG at the age of 4 years was suggestive of mild cortical dysfunction and nonspecific encephalopathy. Another EEG was done at the age of 11 years old and was suggestive of mild cortical dysfunction and nonspecific encephalopathy. Her last EEG at the age of 13 years was normal	At the age of 40 days, EEG was severely abnormal with multiple cortical neuro irritability, indicating a predisposition for epileptic seizures. At 7 years old, the EEG was abnormal and suggestive of mild cordial dysfunction and nonspecific encephalopathy (showed significant improvement after the first EEG). At the age of 8 years old, the EEG was normal
Dental anomaly		Hypodontia, teeth hypoplasia, widely spaced teeth, gingival hyperplasia	Hypodontia, teeth hypoplasia, widely spaced teeth, gingival hyperplasia	Hypodontia, teeth hypoplasia, widely spaced teeth, gingival hyperplasia	Hypodontia, teeth hypoplasia, widely spaced teeth, gingival hyperplasia	Hypodontia, teeth hypoplasia, widely spaced teeth, gingival hyperplasia	Hypodontia, teeth hypoplasia, widely spaced teeth, gingival hyperplasia
Feeding difficulties		(−)	NA	(+)	NA	NA	(+)
Ophthalmology problems					Divergent squint	Exotropia	
Biopsy		Skin biopsy did not show any abnormal changes	NA	NA	NA	Muscle biopsy showed nonspecific type II muscle atrophy	Skin biopsy showed membrane-bound curvilinear-like inclusions are noted in some fibroblasts
Others						Microcephaly	

Abbreviations: EEG, electroencephalogram; MRI, magnetic resonance imaging; MRS: magnetic resonance spectroscopy; NA: not available.

#### Patient 6 (family 3, II:5)

The patient is a 12-year-old boy (Figure 1A). He was diagnosed with familial autosomal recessive epileptic encephalopathy, intractable seizures, and global developmental delay. His seizures started on the first day of his life. At the age of 40 days, his EEG was severely abnormal, with multiple cortical neuroirritability, indicating predisposition for epileptic seizures. At 7 years of age, the EEG was still abnormal and suggestive of mild cordial dysfunction and nonspecific encephalopathy but showed significant improvement compared to the first. The last EEG was performed at the age of 8 years and showed normal results. The patient is still having frequent seizures in addition to his global developmental delay, but from the developmental perspective, he is improving gradually.

### Dental and oral manifestation findings

**Figure 3:**
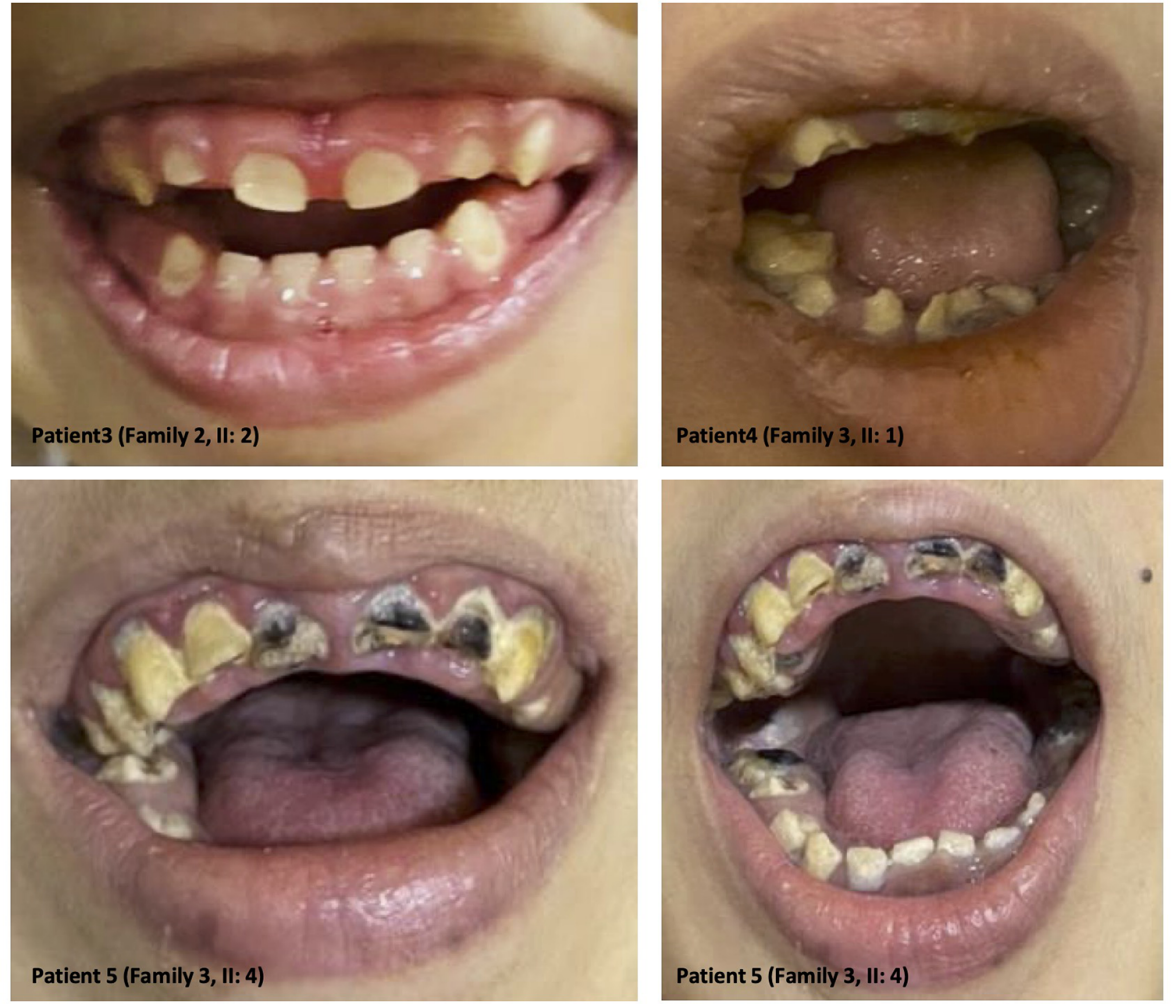
Dental anomalies: illustrative images from the selected patients showing teeth defects and malformations in both groups of young patients with primary teeth and older patients with permanent teeth, as well as open anterior-skeletal bite and overgrowth of the gingiva.

The clinical features of our six patients include not only early-onset epilepsy and delayed neurological development but also significant defects in tooth development such as hypodontia, teeth hypoplasia, widely spaced teeth, and gingival hyperplasia, as we see in amelogenesis imperfecta cases (i.e., the enamel is abnormally thin, soft, fragile, pitted, and discolored), causing severe embarrassment, eating difficulties, and pain in patients. These dental deformities are very difficult to treat as they involve both dental and skeletal deformities. Interestingly, we see teeth defect and malformation in both groups of young patients with primary teeth and older patients with permanent teeth, as well as open anterior-skeletal bite and overgrowth of gingiva (mostly due to poor oral hygiene and the side effect of antiepileptic medications) (Figure 3).

### Genetic findings

Whole exome sequencing (WES) combined with single nucleotide polymorphism (SNP) analysis revealed two homozygous variants of *SLC13A5* as the only strong candidates underlying the disorder. The gene is in a major run of homozygosity (ROH) block detected on the short arm of chromosome 17 in family 1 (Figure 1B). Patients 1, 2, and 3 harbor a previously known single nucleotide insertion (c.1227dupC) (12). The c.1227dupC produces a frameshift, leading to a premature stop codon (*p*.Ile410Hisfs*13). The other variant, a novel donor splice site variant of *SLC13A5* (c.1437 + 5G > A), was identified in the remaining three patients 4, 5, and 6 (family 3) by WES. This variant was absent in local and international databases. Segregation of the variants in the families was performed using Sanger sequencing (Figure 1C).

MutationTaster and varSEAK were used to predict the pathogenicity of c.1227dupC (*p*.Ile410Hisfs*13) and c.1437 + 5G > A, respectively. MutationTaster predicted the variant to be deleterious, leading to a premature stop codon. Moreover, this variant is already reported in ClinVar as a disease-causing variant. On the other hand, varSEAK computed that c.1437 + 5G > A will have a class 5 splicing effect (an exon skipping), resulting in loss of function due to aberrant splicing. Details of these predictions and analysis results are presented in Supplementary File S1.

## Discussion

Epileptic encephalopathies are broad and highly heterogeneous epileptic disorders characterized by seizures and abnormal EEG coupled with cognitive and developmental impairment. Biallelic mutations in *SLC13A5* cause one such disorder called as “developmental and epileptic encephalopathy 25 with amelogenesis imperfecta” (DEE25; phenotype MIM: 615905). In this study, we focused on pathogenic variants of *SLC13A5* in our population and identified six patients from three different Saudi families harboring two homozygous variants (a previously reported insertion and a novel splice site variant). Previously reported patients exceeded 100 cases with approximately 36 different variants (Supplementary Table S1). All these appear to be autosomal recessive, with several families having more than one affected child. Most of the variants are missense (*n* = 24) (13). Additionally, six splicing mutations (13–21), four small deletions (18, 20, 22–26), two small insertions (6, 12, 13, 16, 27, 28), and four gross deletions (13, 20, 29) have been reported.

These pathogenic variants have been linked to various phenotypes, especially seizure-causing syndromes such as Kohlschütter–Tönz and West syndromes (19, 26). However, the current understanding of these syndromes, as well as dissecting the genetic and clinical heterogeneity of developmental and epileptic encephalopathies, revealed that different genes are involved in these syndromes. For example, mutations in *ROGDI* lead to Kohlschütter–Tönz syndrome (MIM: 226750; OMIM: 614574). In children SLC13A5-related early-onset epilepsy, which usually occurs within the first few weeks after birth, seizures continue as the children grow (3, 4). This is thought to be related to loss-of-function mutations. Such mutations cause an inability to transport citrate across the plasma membrane, leading to distinctive symptoms. Affected patients may exhibit physical and cognitive impairment, major delays in speech development, and characteristic dental problems (3, 4, 20). Of note, *SLC13A5* is expressed in a wide range of tissues (22); most of the phenotypes observed in various affected individuals seem related to its expression in the brain. The patients presented with encephalopathy, GDD, and seizures, which appeared during early infancy and mostly began in the first 24 h of life. The electroencephalogram (EEG) recordings also showed multiple abnormalities. Such anomalies were also seen in previously reported patients. For example, Klotz et al. (18) reported nine patients from six different families; all had seizures from the first week of life except for one who experienced a delay in her first seizure at the age of months. Moreover, all of them were noticed to have motor and language delays.

Interestingly, some studies have reported defects in tooth development, such as hypodontia. Such symptoms are considered one of the key characteristics of “developmental and epileptic encephalopathy-25 (DEE25) disorder” (3, 6, 18, 29). It is noteworthy that the pioneering study by Thevenon et al. (3) did not report any major defects of tooth development except for widely spaced teeth. *SLC13A5* has been found to have a crucial role in tooth development, where a gene expression analysis showed *SLC13A5* to be one of the top 15 genes dysregulated during mouse molar development (6). Such involvement caused confusion due to the overlapping clinical features and manifestations between Kohlschütter–Tönz syndrome and DEE25. Moreover, clinical heterogeneity appeared in the disease expression, even between siblings with identical genotypes. For example, a brother and sister carrying the same compound heterozygous variants of *SLC13A5* presented with varying intractable seizures and focal brain lesions; one of the patients had focal cortical dysplasia and was more developmentally delayed than the other sibling. Nevertheless, both had seizures within the first day of life, hypotonia, and developmental delay (30).

The earliest study identified *SLC13A5* variants as the disease-causing variants that reported overlapping clinical features such as early-onset seizures, which appeared within any day of the first week of life, with severe developmental delay. In addition to seizures that may even appear as early as on the first day of life, there were other phenotypes, such as pediatric movement disorders and intellectual disability, linked to pathogenic variants of *SLC13A5* (6, 14, 31).

The six affected patients in our study have the characteristics mentioned before, with predominant GDD and epilepsy. The other features observed in our patients are as follows: patients 1 and 5 also presented with microcephaly at early stages of life; patients 1 and 3 had spasticity; patients 3 and 5 had speech problems; patients 1, 2, 3, and 5 presented with motor difficulties; and finally patient 5 showed muscle atrophy, recurrent skin abscess, and exotropia.

Until now, there is no specific treatment for *SLC13A5*-epileptic encephalopathy. Considering that the seizures are refractory, all our patients are on multiple anticonvulsant medications to control their seizures. Some are on three anticonvulsant medications (levetiracetam, topiramate, and phenobarbital), while others were on topiramate along with valproic acid and diazepam. However, none of the patients have been reported to be effectively weaned off their medications. One of the proposed mechanisms that can help to control seizures in *SLC13A5*-epileptic encephalopathy is using medications that can increase the cytoplasmic level of citrate. This could lead to a decrease in the frequency of seizures and help control the patient's symptoms. Triheptanoinone acts by increasing the metabolism of odd-chain fatty acids in neuronal mitochondria; as a result, it increases the levels of succinyl-CoA, which leads to the increase of citrate concentration. Benzodiazepines are another example. These classes of medications share an augmentation mechanism by enhancing the inhibitory action of gamma aminobutyric acid (GABA) (28). Moreover, other antiepileptic medications can also be used, such as phenytoin or lamotrigine, as these drugs target sodium channels. One of the new-generation drugs that have multiple mechanisms of action to control seizures is stiripentol. One of its action mechanisms is based on enhancing GABAergic transmission. Stiripentol can also be used as an adjunctive therapy to valproate and clobazam. Moreover, acetazolamide, a carbonic anhydrase inhibitor and an atypical medication, can also suppress seizures by an unknown mechanism of action. On the other hand, nonpharmacological treatments such as ketogenic diet have been reported to reduce seizure frequency, although their efficiency is controversial (28). Still, there is a need to investigate new therapeutic targets and more effective treatments for this disorder in the future.

In conclusion, we reported a novel homozygous variant of *SLC13A5* mutation in a previously unstudied population for this disease. Given the growing number of epilepsy genes linked to early-onset epileptic encephalopathies, WES and gene panels can improve the identifying known and unknown pathogenic variants in the gene. Although there is currently no curative treatment available for the disease, early detection of the deleterious variants may provide an advantage in terms of premarital screening, genetic counseling, and avoiding unnecessary and redundant diagnostic tests and treatments. Improving seizure control should also be one of the components of future therapeutic trials and research.

## Data Availability

The original contributions presented in the study are included in the article and Supplementary Material, further inquiries can be directed to the corresponding author.
